# Association of Perceived Xingfu With Health-Related and Socioeconomic Factors Among Hong Kong Chinese Adults: Cross-Sectional Study Using a Novel Single-Item Tool

**DOI:** 10.2196/73350

**Published:** 2025-07-07

**Authors:** Katherine Y P Sze, Sai Yin Ho, Agnes Yuen Kwan Lai, Jing Jia, Heng Xu, Shirley Man Man Sit, Tai Hing Lam, Man Ping Wang

**Affiliations:** 1 School of Nursing, University of Hong Kong, 5/F, HKUMed Academic Building, 3 Sassoon Road, Pokfulam, Hong Kong, China (Hong Kong), 852 39176636; 2 School of Public Health, University of Hong Kong, Hong Kong, China (Hong Kong); 3 School of Nursing and Health Studies, Hong Kong Metropolitan University, Hong Kong, China (Hong Kong); 4 Department of Social and Behavioural Sciences, Department of Social and Behavioural Sciences, City University of Hong Kong, Hong Kong, China (Hong Kong)

**Keywords:** perceived xingfu (幸福感), health-related factors, socioeconomic factors, Hong Kong, cross-sectional study

## Abstract

**Background:**

Xingfu (幸福), a Chinese term, holds meanings that transcend Western concepts of happiness; it is modern and highly valued in China. Despite its centrality to China’s national discourse on xingfu, there are no validated tools for measuring perceived xingfu, particularly in Hong Kong’s unique sociopolitical context. Postpandemic recovery efforts and widening socioeconomic disparities in Hong Kong highlight the urgency of understanding indicators such as perceived xingfu.

**Objective:**

This study aimed to develop and validate the first single-item measure of perceived xingfu and examine its association with health-related and socioeconomic factors among Hong Kong Chinese adults, addressing gaps in culturally tailored assessment.

**Methods:**

Our cross-sectional online survey included 5070 Hong Kong Chinese adults in 2023. Perceived xingfu was measured using a novel, single-item, 11-point scale from 0 to 10, with higher scores indicating better perceived xingfu. Two-week test-retest showed high reliability (intraclass correlation coefficient of 0.78). We used regression models to analyze associations between perceived xingfu score and high perceived xingfu (defined as ≥7) with mutually adjusted study variables; all estimates were weighted based on 2022 Hong Kong population data.

**Results:**

The mean perceived xingfu score was 6.3 (SD 2.2). Perceived xingfu score was associated with happiness (*r*=0.85), perceived mental health (*r*=0.65), and adversity coping capability (*r*=0.50) and negatively associated with perceived stress (*r*=−0.56), past 7-days loneliness (*r*=−0.52), anxiety symptoms (*r*=−0.45), and depressive symptoms (*r*=−0.52). Female sex (β=0.69, adjusted odds ratio [aOR] 2.11), older age (β=0.46, aOR 2.67), having post-secondary education or above (β=0.19, aOR 1.35), higher monthly household income (≥ HKD60,000: β=0.99, aOR 3.04), living in owned properties (β=0.27, aOR 1.57), being retired (β=0.56, aOR 1.18), and excellent versus poor self-rated health (*β*=3.84, aOR 40.72) were associated with higher perceived xingfu score or high perceived xingfu (all *P*s or *P*s_trend_<0.001).

**Conclusions:**

This study pioneers the perceived xingfu measurement in a Chinese population using a concise, validated tool. Significant socioeconomic disparities and health associations highlight perceived xingfu’s relevance to policy priorities, including equitable resource allocation and health support. Our single-item perceived xingfu tool offers practical utility for population surveillance and cross-cultural comparisons. Future research should explore longitudinal trends and integrate perceived xingfu into public health frameworks.

## Introduction

The meanings of xingfu are perceived as transcending Western concepts or constructs such as happiness, mental health, well-being, quality of life, and life satisfaction [1]. The term xingfu, in Chinese (幸福), has been commonly used by intellectuals and politicians since the early years of modern China. Multimedia Appendix 1 shows the cultural relevance and development of the concept of xingfu. Xingfu is more complex than happiness and belongs to a higher level of human and political pursuit [2]. Since the establishment of the People’s Republic of China in 1949, enhancing the xingfu of the population has been elevated to be the most important aim of the government and the Chinese Communist Party. Perceived xingfu has been central to China’s governance since 1949, and has been emphasized as a national priority from Mao-era policies to the current “great rejuvenation” agenda [3,4]. In vernacular Chinese, the terms xingfu and happiness are often used together, suggesting that the two are closely connected but distinct constructs. Xingfu and happiness have been referenced in various studies and speeches, which show the connections and differences [5,6]. Despite its sociopolitical prominence, there is no consensus on the definition or measurement of xingfu or original research on xingfu and perceived xingfu among Chinese people. Direct perceived xingfu measurement remains undeveloped due to definitional ambiguities and over-reliance on Western models.

Xingfu (幸福) is often translated to “happiness” in English [7]. Conventionally, “happiness” is translated to “快樂” or “幸福” in Chinese, but “快樂” is translated into “happiness,” “pleasure,” or “enjoyment;” however, these 3 English words do not mean xingfu. The World Happiness Report translated to “世界幸福報告” or “世界快樂報告,” with “happiness” being translated to 幸福 (xingfu) or 快樂 (happiness) but not both, has no measures on subjective happiness nor xingfu [8]. The Chinese construct of xingfu does not have a direct counterpart in Western paradigms although “well-being” is translated into “康樂,” “安康,” “健康,” “福祉,” “幸福” etc. in Chinese, indicating well-being has different meanings and is not xingfu. Despite their interconnectedness, xingfu, happiness, and well-being are distinct constructs, including “objective” indicators and subjective feelings or perceptions. Happiness, as a subjective construct, is associated with physical and mental health [9,10], but the associations of self-reported or perceived xingfu (幸福感) with health-related and socioeconomic factors remain unclear.

The 2023 Policy Address of the Hong Kong Special Administrative Region (HKSAR) Chief Executive marked the first use of “xingfu” as the headline, and introduced initiatives to promote the residents’ xingfu [11]. This shows that the HKSAR Government has started to prioritize promoting xingfu, which was rarely mentioned in Hong Kong under the British rule before 1997. Hong Kong is one of the most westernized cities in China, with 91.6% of the population being ethnic Chinese [12]. Whether people in Hong Kong or Chinese communities living elsewhere can perceive and report xingfu is unknown. This study aimed to measure perceived xingfu using our newly developed single-item tool and analyze its association with health-related and socioeconomic factors in Hong Kong Chinese adults.

## Methods

### Survey Design

We conducted a cross-sectional survey in 2023 to measure perceived xingfu and analyze its association with health-related and socioeconomic factors in 5070 Chinese individuals aged 18 years or older, recruited from an online nonprobabilistic panel maintained by the Hong Kong Public Opinion Research Institute, a locally well-known survey agency. Invitations were distributed through email and the respondents voluntarily completed the online questionnaires, with reminders sent twice monthly. Of the 79,456 eligible respondents, 5070 completed the entire survey, with a response rate of 6.4%. This study adhered to the Checklist for Reporting Results of Internet E-Surveys (CHERRIES) for reporting guidelines [13]. Data were weighted by sex, age, educational attainment, and economic activity status based on the 2022 Hong Kong population data.

### Ethical Considerations

Data confidentiality was maintained through deidentification of all respondents’ records. Survey responses were stored on a password-protected server accessible only to the research team. Participants provided informed consent electronically prior to survey access. A small monetary incentive was given for questionnaire response and no images or personal identifiers are included in this study. The Institutional Review Board of the University of Hong Kong/Hospital Authority Hong Kong West Cluster granted ethical approval (UW 20‐651) for this survey.

### Instruments

We measured perceived xingfu by asking the question “你認為你有多幸福?” (“How xingfu do you think you are?”) with responses on a scale of 0‐10, with higher scores indicating higher levels of perceived xingfu. We conducted reliability testing on perceived xingfu in 147 Hong Kong residents. The two-week test-retest intraclass correlation coefficient was 0.78. This single-item perceived xingfu scale was validated and minimizes respondent burden in population-based surveys. Psychosocial factors, measured on a scale of 0‐10, included personal happiness, perceived mental health, and adversity coping capability (ACC), with higher scores indicating higher levels. Self-rated health was measured on a five-point scale, ranging from poor to excellent [14]. Respondents also reported the number of days they felt lonely in the past 7 days, on a scale ranging from 0 to 7 days [15]. Anxiety and depressive symptoms were measured using the Patient Health Questionnaire-4. The Chinese subscales that measure anxiety and depressive symptoms had Cronbach’s α coefficients of 0.82 and 0.79, respectively [16]. Perceived stress was measured using the validated Perceived Stress Scale-4, with higher scores indicating greater stress levels [17]. Information on socioeconomic factors, including sex, age, education level, monthly household income, housing, and employment status, was also collected.

### Statistical Analysis

We used descriptive statistics for socioeconomic factors, self-rated health, loneliness, anxiety and depressive symptoms, and psychosocial factors. Pearson correlation coefficients were used to examine correlations between perceived xingfu and psychosocial factors, treating both as continuous variables. Linear regression models were used to examine the associations between socioeconomic factors, self-rated health, and perceived xingfu scores using adjusted β coefficients and 95% CIs. Multivariable logistic regression analyses were used to estimate the adjusted odds ratios (aORs) and 95% CIs of high perceived xingfu (defined as ≥7) in relation to socioeconomic factors and self-rated health. The β coefficients and odds ratios (ORs) were mutually adjusted. Variance inflation factor (VIF) showed low multicollinearities for all predictors (VIFs <2). All statistical analyses were conducted using the STATA software (version 15.0; StataCorp). A *P* value <.05 was considered statistically significant.

## Results

Table 1 shows that in the weighted sample, 53.2% (n=2390) were female, 64.0% (n=2811) were aged 35‐64, 63.0% (n=2852) had secondary or lower education, 50.6% (n=1938) had a household monthly income of HKD20,000‐59,999 (1 USD=7.8HKD), 60.1% (n=2570) lived in owned properties, and 58.9% were employed. Additionally, 33.7% (n=1510) self-rated their health as fair and 33.7% (n=1520) as good, while 51.9% (n=2219) reported no loneliness in the past 7 days. Moreover, 17.4% (n=752) and 17.8% (n=775) reported anxiety and depressive symptoms, respectively. The mean scores for psychosocial variables were as follows: perceived xingfu, 6.3 (SD 2.2); perceived stress, 6.6 (SD 3.2); perceived mental health, 6.3 (SD 2.2); personal happiness, 5.8 (SD 2.2); and ACC) 6.3 (SD 1.9).

**Table 1.** Socioeconomic characteristics of survey sample of Hong Kong Chinese adults.

Variables	Unweighted (n=5070)	Weighted sample (n=4515)^a^
Sex, n (%)		
Male	2213 (49.0)	2101 (46.8)
Female	2302 (51.0)	2390 (53.2)
Age group (years), n (%)		
18‐34	1556 (34.7)	920 (20.6)
35‐64	2624 (58.5)	2811 (63.0)
≥65	306 (6.8)	732 (16.4)
Education, n (%)		
Secondary or below	702 (15.7)	2852 (64.0)
Post-secondary or above	3776 (84.3)	1603 (36.0)
Monthly household income,^b^ n (%)		
≤ HKD 19,999	556 (14.2)	1001 (26.2)
HKD 20,000‐59,999	1938 (49.4)	1938 (50.6)
≥ HKD 60,000	1431 (36.5)	889 (23.2)
Housing, n (%)		
Rented	1669 (38.6)	1706 (40.0)
Owned	2659 (61.4)	2570 (60.1)
Employment status, n (%)		
Employed	3422 (76.9)	2608 (58.9)
Unemployed	528 (11.9)	727 (16.4)
Retired	502 (11.3)	1096 (24.7)
Self-rated health, n (%)		
Poor	156 (3.1)	173 (3.9)
Fair	1545 (30.5)	1510 (33.7)
Good	1703 (33.7)	1520 (33.7)
Very good	1471 (29.1)	1161 (25.9)
Excellent	184 (3.6)	124 (2.8)
Past 7-days loneliness (days), n (%)		
0	2323 (47.8)	2219 (51.9)
1‐2	1623 (33.4)	1257 (29.4)
3‐4	542 (11.2)	475 (11.1)
5‐6	194 (4.0)	134 (3.1)
7	174 (3.6)	187 (4.4)
Patient Health Questionnaire-4 anxiety symptoms, n (%)		
No	3958 (80.1)	3572 (82.6)
Yes	983 (19.9)	752 (17.4)
Patient Health Questionnaire-4 depressive symptoms, n (%)		
No	4017 (80.9)	3569 (82.2)
Yes	948 (19.1)	775 (17.8)
Psychosocial variables, mean (SD)		
Perceived xingfu (perceived xingfu; 幸福感) score, range from 0‐10	6.4 (2.1)	6.3 (2.2)
Perceived Stress Scale-4 score, range from 0‐16	6.9 (3.2)	6.6 (3.2)
Perceived mental health score, range from 0‐10	6.2 (2.2)	6.3 (2.2)
Personal happiness score, range from 0‐10	5.9 (2.1)	5.8 (2.2)
Adversity coping capability score, range from 0‐10	6.4 (1.9)	6.3 (1.9)

a Missing data (9%) were excluded. Data were weighted by sex, age, education attainment, and economic activity status based on 2022 Hong Kong population data.

b 1 USD =7.8 HKD.

Table 2 shows that perceived xingfu was strongly and positively correlated with personal happiness (*r*=0.85), perceived mental health (*r*=0.65), and ACC (*r*=0.50); perceived xingfu was negatively correlated with perceived stress (*r*=−0.56), past 7-day loneliness (*r*=−0.52), anxiety symptoms (*r*=−0.45), and depressive symptoms (*r*=−0.52; all *P*s<0.001).

**Table 2.** Correlations of perceived xingfu (幸福感) with psychosocial factors^a^.

	Perceived xingfu	PSS-4^b^	Past 7-day loneliness	Perceived mental health	Happiness	ACC^c^	PHQ-4^d^ anxiety symptoms
PSS-4	−0.56	–^e^	–	–	–	–	–
Past 7-day loneliness	−0.52	0.52	–	–	–	–	–
Perceived mental health	0.65	−0.67	−0.57	–	–	–	–
Personal happiness	0.85	−0.60	−0.54	0.71	–	–	–
ACC	0.50	−0.56	−0.37	0.61	0.54	–	–
PHQ-4 anxiety symptoms	−0.45	0.62	0.54	−0.64	−0.52	−0.4	–
PHQ-4^d^ depressive symptoms	−0.52	0.66	0.61	−0.66	−0.58	−0.4	0.72

a Missing data (9%) were excluded. Data were weighted by sex, age, education attainment and economic activity status based on 2022 Hong Kong population data. Higher scores indicating higher levels for all variables. All *P* values for Pearson correlation coefficients <0.001.

b PSS-4: Perceived Stress Scale-4.

c ACC: adversity coping capability.

d PHQ-4: Patient Health Questionnaire-4.

e Not applicable.

**Figure 1.**
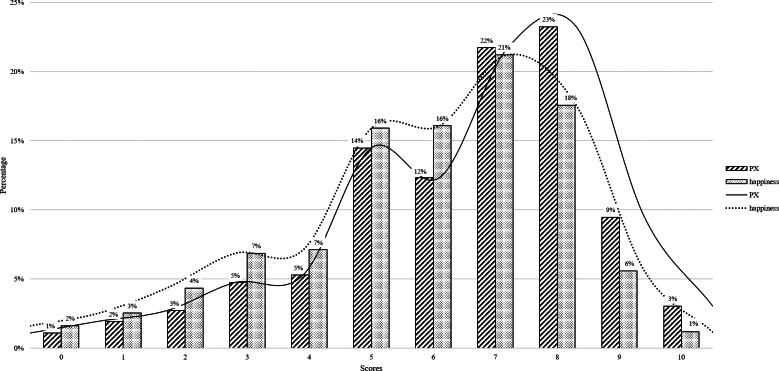
Distribution of perceived xingfu and happiness scores. PX: perceived xingfu.

Figure 1 shows the distribution of perceived xingfu and happiness scores. As both perceived xingfu and happiness peaked at scores of 7 (22%) and 8 (23%); therefore, perceived xingfu ≥7 was classified as high perceived xingfu in the logistic regression model. Similar perceived xingfu and happiness scores were reported by 48.9% (n=2478) of respondents, while 46.5% (n=2360) showed a difference between perceived xingfu and happiness scores ranging from −1 to +3. The remaining 4.6% (n=232) showed a difference greater than −1 and less than +3 (Multimedia Appendix 1).

Table 3 shows that, in the linear regression model, the following variables were associated with higher perceived xingfu scores: being female (β=0.69, 95% CI 0.58-0.81; *P<*.001); being aged 35-64 years (β=0.23, 95% CI 0.10-0.36) or ≥65 years (β=0.46, 95% CI 0.15-0.76; *P*_trend_<.001); having post-secondary education or above (β=0.19, 95% CI 0.02-0.36; *P<*.01); having a higher household monthly income (HKD20,000‐59,999: β=0.33, 95% CI 0.14-0.52; ≥ HKD60,000: β=0.99, 95% CI 0.79-1.20; *P*_trend_<.001); living in owned properties (β=0.27, 95% CI 0.14-0.39; *P<*.001); being retired (β=0.56, 95% CI 0.33-0.79; *P<*.001); and having excellent versus poor self-rated health (β=3.84, 95% CI 3.39‐4.29; *P<*.001).

Table 3 also shows that in the logistic regression model, the following variables were associated with higher odds of reporting high perceived stress (≥7): female sex (aOR 2.11, 95% CI 1.82‐2.46; *P<*.001); those aged 35‐64 (aOR 1.64, 95% CI 1.34‐2.02) or ≥65 (aOR 2.67, 95% CI 1.97‐3.62; *P_trend_*<.001), respondents with post-secondary education or above (aOR 1.35, 95% CI 1.14‐1.59; *P<*.001), higher household monthly income (HKD20,000‐59,999: aOR 1.74, 95% CI 1.45‐2.09;≥ HKD60,000: aOR 3.04, 95% CI 2.41‐3.83; *P*_trend_<.001), and those living in owned properties (aOR 1.57, 95% CI 1.34‐1.83, *P<*.001). Respondents who reported better self-rated health had higher odds of high perceived xingfu (*P*_trend_<.001), with the largest aOR in those who reported excellent health (aOR 4.72, 95% CI 18.43‐89.93; *P<*.001).

**Table 3.** Linear and logistic regressions of perceived xingfu (幸福感) with socioeconomic factors and self-rated health.

Variables^a^	Perceived xingfu, mean (SD)	β (95% CI)^b^	*P* value^c^	High levels of perceived xingfu (≥7), n (%)	aOR^d^ (95% CI)^b^	*P* value^c^
Sex			<.001			<.001
Male	6.0 (2.2)	0		1008 (48.0)	1	
Female	6.5 (2.1)	0.69 (0.58- 0.81)		1449 (6.6)	2.11 (1.82‐2.46)	
Age group (years)			<.001^f^			<.001^f^
18‐34	6.0 (2.2)	0		439 (47.7)	1	
35‐64	6.3 (2.2)	0.23 (0.10-0.36)	<.001	1536 (54.7)	1.64 (1.34‐2.02)	<.001
≥65	6.8 (1.8)	0.46 (0.15-0.76)	<.001	464 (63.4)	2.67 (1.97‐3.62)	<.001
Education			.001			<.001
Secondary or below	6.1 (2.2)	0		1462 (51.3)	1	
Post-secondary or above	6.6 (2.0)	0.19 (0.02-0.36)		976 (6.9)	1.35 (1.14‐1.59)	
Monthly household income^e^			<.001^f^			<.001^f^
≤ HKD19,999	5.9 (2.3)	0		436 (43.5)	1	
HKD20,000‐59,999	6.3 (2.1)	0.33 (0.14-0.52)	<.001	1066 (55.0)	1.74 (1.45‐2.09)	<.001
≥ HKD60,000	7.1 (1.7)	0.99 (0.79‐1.20)	<.001	632 (71.1)	3.04 (2.41‐3.83)	<.001
Housing			<.001			<.001
Rented	5.8 (2.3)	0		755 (44.3)	1	
Owned	6.6 (2.0)	0.27 (0.14-0.39)		1573 (61.2)	1.57 (1.34‐1.83)	
Employment status			<.001^f^			.16^f^
Employed	6.2 (2.1)	0		1817 (53.1)	1	
Unemployed	5.8 (2.5)	0.01 (–0.19 to 0.21)	.843	245 (46.5)	1.05 (0.84‐1.31)	.649
Retired	6.9 (1.8)	0.56 (0.33-0.79)	<.001	324 (64.5)	1.18 (0.95‐1.45)	.131
Self-rated health			<.001^f^			<.001^f^
Poor	3.7 (2.5)	0		26 (14.9)	1	
Fair	5.5 (2.1)	1.61 (1.26‐1.97)	<.001	563 (37.3)	3.19 (1.89‐5.40)	<.001
Good	6.6 (1.9)	2.34 (1.99‐2.69)	<.001	907 (59.7)	7.31 (4.32‐12.35)	<.001
Very good	7.2 (1.8)	2.95 (2.60‐3.31)	<.001	854 (73.6)	14.21 (8.35‐24.18)	<.001
Excellent	8.1 (1.8)	3.84 (3.39‐4.29)	<.001	105 (85.0)	4.72 (18.43‐89.93)	<.001

a Missing data (9%) were excluded.

b Mutually adjusted.

c Data were weighted by sex, age, education attainment and economic activity status based on 2022 Hong Kong population data.

d aOR: adjusted odds ratio.

e USD=7.8 HKD.

f *P* for trend.

## Discussion

### Principal Findings

We first developed a novel, single-item question to measure perceived xingfu and showed that xingfu is a unique psychosocial construct in Chinese culture. Our findings show that perceived xingfu is strongly associated with socioeconomic, psychosocial, and health-related factors, aligning with—but also diverging from—Western concepts of happiness. Strong positive correlations were observed between perceived xingfu and perceived mental health, personal happiness, and ACC, while negative correlations with negative constructs, including perceived stress, past 7-day loneliness, anxiety and depressive symptoms demonstrate the multidimensionality of this instrument.

Socioeconomic factors, including being female, older age, higher education level, higher monthly household income, and living in owned properties, were positively associated with higher perceived xingfu scores and high levels of perceived xingfu. These findings align with global patterns in which socioeconomic advantages are exacerbated with higher levels of happiness; however, Hong Kong’s extreme income inequality and housing scarcity likely increase these disparities. For instance, the steep income gradient (ie, higher aORs with increasing income) shows the significant role of financial security in high-cost environment, while homeownership reflects the importance of psychological safety gained by property ownership in a volatile housing market [18,19]. Such socioeconomic disparities in perceived xingfu are expected, as xingfu is strongly correlated with happiness. Interestingly, retired respondents reported higher perceived xingfu scores than employed respondents, which contrasts with previous studies on employment and life satisfaction [20]. This may reflect cultural values in Hong Kong, where retirement is associated with familial respect, reduced work-related stress, or financial preparedness through systems [21]. Future studies could investigate how younger generations prioritize career success, while older generations place greater emphasis on family harmony and traditional values. Higher perceived xingfu among females and older adults also aligns with Western findings of increased well-being in these groups [22], while strong social networks or Confucian norms may mediate these outcomes in Chinese societies [23]. While these findings differ from or are in parallel with Western research on well-being, happiness, life satisfaction, and quality of life, perceived xingfu remains a culturally distinct construct in Chinese societies. Thus, caution is needed when generalizing these findings. Policy efforts targeting income inequality, housing access, and health equity could enhance perceived xingfu; however, interventions must consider Hong Kong’s unique sociocultural environment.

Although xingfu has been a less commonly used construct in Hong Kong than in Mainland China, respondents had no difficulty in answering our question and giving a score. Moreover, the mean perceived xingfu score was higher than the mean happiness score, with only 51% reporting the same score. This suggests perceived xingfu transcends transient emotional states of happiness, which aligns with prior studies emphasizing on xingfu as a holistic, socially anchored concept in Chinese societies, distinct from Western individualism-centric metrics [2]. Culture-specific measures rather than direct translations of Western instruments are needed. Direct translations of Western tools pose a risk of overlooking contextual priorities, such as the emphasis on familial cohesion over personal achievement among older generations. Policies and measures to promote xingfu or perceived xingfu to the populations or communities must also be capable of reducing disparities. People of different sexes, ages, and socioeconomic statuses may have different needs and perceptions, which warrantees further studies. By focusing on factors that may influence perceived xingfu within Hong Kong’s sociopolitical and cultural context, our study can contribute to a deeper understanding of perceived xingfu as a unique psychosocial construct in Chinese societies. These findings indicate that perceived xingfu represents a more nuanced construct than happiness alone. Hence, perceived xingfu should be measured and monitored in populations and communities where xingfu is highly valued, either beyond or together with happiness. Experiences and achievements in promoting perceived xingfu in Hong Kong and Mainland China could be better understood and disseminated to other countries and regions.

### Limitations

Our study has some limitations. First, perceived xingfu and xingfu may represent different meanings or domains and are susceptible to cultural nuances and contextual factors. Qualitative research is needed to provide deeper insights into the multidimensional nature of perceived xingfu and xingfu. Second, although one of the Chinese translations of “well-being” is 幸福, we did not analyze well-being and perceived xingfu, because of the lack of commonly accepted and simple tools for measuring subjective well-being. Third, we measured perceived xingfu rather than xingfu as an “objective” construct defined by environmental or often external indicators. Further studies on whether some of the domains or indicators included in the World Happiness Report may be applicable domains or indicators of xingfu in Chinese contexts, are warranted. Fourth, while online recruitment ensured efficiency, it may underrepresent elderly or low-income populations with limited digital access. Future studies should combine online and community-based sampling to enhance generalizability. Fifth, possible social desirability bias exist due to self-reporting sensitive psychological states. Our tool is simple and should be useful for measuring socioeconomic disparities in the pursuit of xingfu by the government for the people and people’s individual pursuit of xingfu. Hence, monitoring perceived xingfu in the population regularly is both feasible and warranted. If xingfu is to be assessed or measured by different domains indirectly, perceived xingfu should still be included as a direct measure and weighted appropriately. Sixth, the cross-sectional design precludes causal inference. Future studies should compare perceived xingfu across Chinese subpopulations, such as those in Mainland China, Hong Kong, and overseas. Longitudinal studies can offer insights into perceived xingfu’s dynamic nature and potential causal relationships with identified factors.

### Conclusions

Our novel, simple, single-item tool showed that perceived xingfu was associated with socioeconomic, psychological, and health-related factors. Perceived xingfu is a unique Chinese psychosocial construct that frequently appears with, but is distinct from happiness. Monitoring perceived xingfu could inform equitable health policies in Hong Kong and beyond. Further exploration of the applicability of this measurement in different regions of China and among different populations can help assess its generalizability, and provide a more comprehensive understanding of perceived xingfu across various Chinese communities within and beyond China.

## Supplementary material

10.2196/73350 Multimedia Appendix 1 Distribution of perceived xingfu (幸福感) and happiness. Explanation of xingfu (幸福).

10.2196/73350 Checklist 1 Checklist for Reporting Results of Internet E-Surveys (CHERRIES).

## Abbreviations

ACC: adversity coping capability
aOR: adjusted odds ratio
