# Supplementary material for: Association of Perceived Xingfu With Health-Related and Socioeconomic Factors Among Hong Kong Chinese Adults: Cross-Sectional Study Using a Novel Single-Item Tool
Source: JMIR Form Res. 2025 Jul 7;9:e73350. doi: 10.2196/73350 (PMC12284738; doi:10.2196/73350)
Supplement: Multimedia Appendix 1 [file formative-v9-e73350-s001.docx]

**Appendix 1. Distribution of perceived xingfu (PX; 幸福感) and happiness**

Table 1. Responses on PX scores and happiness scores

| Respondent’s response | n | % |
| --- | --- | --- |
| reported same scores | 2478 | 48.88% |
| reported difference scores | 2592 | 51.12% |
| total | 5070 | 100.00% |

Table 2. Difference between PX scores and happiness scores in respondents who reported different scores

| PX scores minus happiness scores | n | % |
| --- | --- | --- |
| -5 | 6 | 0.23% |
| -4 | 5 | 0.19% |
| -3 | 15 | 0.58% |
| -2 | 78 | 3.01% |
| -1 | 414 | 15.97% |
| 1 | 1219 | 47.03% |
| 2 | 529 | 20.41% |
| 3 | 198 | 7.64% |
| 4 | 66 | 2.55% |
| 5 | 32 | 1.23% |
| 6 | 14 | 0.54% |
| 7 | 8 | 0.31% |
| 8 | 3 | 0.12% |
| 9 | 4 | 0.15% |
| 10 | 1 | 0.04% |
| Total | 2592 | 100% |

**Explanation of xingfu (幸福)**

Xingfu (幸福), often translated into “happiness “in English, emerged as a new construct in modern China. No English words have similar meanings to xingfu. Since the May Fourth Movement in 1919 (1), many famous intellectuals and influential political figures have used xingfu with new but different meanings. The meanings of xingfu in Chinese includes but not limited to happiness in English. In pursuit of xingfu, Lu Xun (魯迅) emphasized the pursuit of the spiritual aspect (2), Hu Shi (胡適) advocated individual freedom (3), Chen Duxiu (陳獨秀) argued for fairness and justice in the social system (4), Xu Zhimo (徐志摩) focused on love and family (5), and Lin Yutang (林語堂) promoted the art of living (6). With the establishment of the People's Republic of China in 1949, during Mao Zedong's era, the song “The East Is Red” (東方紅) popularized the concept of xingfu among the Chinese people (7). President Xi Jinping mentioned the concepts of xingfu and happiness together in 2014, indicating xingfu and happiness are two different constructs. The term xingfu has become a prominent construct in the meanings of China (8, 9) and is often used in festival (such as Chinese New Year) and other greetings by Chinese people.

**References**

1. Xu JL. *“五四”的歷史記憶: 什麼樣的愛國主義？* [Historical memory of the May Fourth Movement: What kind of patriotism?]. Read; 2009.
2. Liu HZ. “體貼人情”：魯迅的幸福觀及寫作動力 [“Considering human feelings”: Lu Xun's view of xingfu and writing motivation]. *Literary Criticism*. 2016.
3. Gao LK. 新文化運動中的個人主義 [Individualism in the New Culture Movement]. *Historical Monthly*. 2015.
4. Zhan ZR, Zhang J. 論中國共產黨早期民生思想 [On the early thoughts of the Communist Party of China on people’s livelihood]. *Journal of the Party School of the Hangzhou Municipal Committee of the Communist Party of China*. 2016.
5. Zhang HL. 一個信仰感情的人－解讀徐志摩的愛 [A person who believes in emotions - Interpretation of Xu Zhimo’s love]. *Anhui Literature*. 2010.
6. Lin YT. *吾國與吾民* [Our Country and Our People]. Baowentang Bookstore; 1988.
7. *The East is Red, The Helmsman Sets the Ocean Course, Long Live Chairman Mao –Songs and Music; and songbook*. Beijing: China Record Company; 1967.
8. Liou CS, Ding SF. *China Dreams: China's New Leadership And Future Impacts. World Scientific*; 2015.
9. Yang Guangyu NY. 習言道︱人生幸福快樂, 強身健體十分重要 [To live a xingfu and happy life, it is very important to keep fit]. *China News*. 2023 [cited 2023 Apr 21]. Available from: http://politics.people.com.cn/BIG5/n1/2023/0421/c1001-32670348.html
